# Incidence and risk factors for Preeclampsia in a cohort of healthy nulliparous pregnant women: a nested case-control study

**DOI:** 10.1038/s41598-019-46011-3

**Published:** 2019-07-02

**Authors:** Jussara Mayrink, Renato T. Souza, Francisco E. Feitosa, Edilberto A. Rocha Filho, Débora F. Leite, Janete Vettorazzi, Iracema M. Calderon, Maria H. Sousa, Maria L. Costa, Philip N. Baker, Jose G. Cecatti, Preterm SAMBA study group, Mary A. Parpinelli, Karayna G. Fernandes, José P. Guida, Danielly S. Santana, Ricardo M. Barbosa, Rafael B. F. Galvao, Bianca F. Cassettari, Lucia Pfitscher, Daisy Lucena de Feitosa, Elias Melo Ferreira Júnior, Danilo Anacleto, Vilma Zotareli, Marcia Alice Silva

**Affiliations:** 1 Department of Obstetrics and Gynaecology, University of Campinas (UNICAMP) School of Medical Sciences, Campinas, SP Brazil; 2 MEAC – Maternity School of the Federal University of Ceará, Fortaleza, CE Brazil; 3 Department of Maternal and Child Health, Maternity Hospital, Federal University of Pernambuco, Recife, PE Brazil; 4 Department of Obstetrics and Gynaecology, Maternity Hospital, Federal University of RS, Porto Alegre, RS Brazil; 5 Department of Obstetrics and Gynaecology, Botucatu School of Medicine, Unesp, Botucatu, SP Brazil; 6 Statistics Unit, Jundiai School of Medicine, Jundiaí, SP Brazil; 7 College of Life Sciences, Maurice Shock Building, University of Leicester, Leicester, UK

**Keywords:** Medical research, Risk factors

## Abstract

The objective of this study is to determine the incidence, socio-demographic and clinical risk factors for preeclampsia and associated maternal and perinatal adverse outcomes. This is a nested case-control derived from the multicentre cohort study Preterm SAMBA, in five different centres in Brazil, with nulliparous healthy pregnant women. Clinical data were prospectively collected, and risk factors were assessed comparatively between PE cases and controls using risk ratio (RR) (95% CI) plus multivariate analysis. Complete data were available for 1,165 participants. The incidence of preeclampsia was 7.5%. Body mass index determined at the first medical visit and diastolic blood pressure over 75 mmHg at 20 weeks of gestation were independently associated with the occurrence of preeclampsia. Women with preeclampsia sustained a higher incidence of adverse maternal outcomes, including C-section (3.5 fold), preterm birth below 34 weeks of gestation (3.9 fold) and hospital stay longer than 5 days (5.8 fold) than controls. They also had worse perinatal outcomes, including lower birthweight (a mean 379 g lower), small for gestational age babies (RR 2.45 [1.52–3.95]), 5-minute Apgar score less than 7 (RR 2.11 [1.03–4.29]), NICU admission (RR 3.34 [1.61–6.9]) and Neonatal Near Miss (3.65 [1.78–7.49]). Weight gain rate per week, obesity and diastolic blood pressure equal to or higher than 75 mmHg at 20 weeks of gestation were shown to be associated with preeclampsia. Preeclampsia also led to a higher number of C-sections and prolonged hospital admission, in addition to worse neonatal outcomes.

## Introduction

Preeclampsia is considered an important cause of maternal mortality and severe maternal morbidity^1^. For every woman who dies, it is estimated that around 20 other women suffer from severe morbidity and disability^2,3^. In view of the social and economic implications of this condition, great effort has been made to expeditiously prevent, diagnose and treat preeclampsia^4–7^.

The magnitude of the problem in some places across the world is still not fully known, especially in low and middle-income countries. In particular, the actual incidence of preeclampsia remains largely unknown^8^. There is usually suboptimal reporting of the disease, leading to constraints on public health applicability^9^. Another important aspect is the identification of pregnant women at risk of developing preeclampsia, especially in nulliparous women with no track record of any pregnancy outcomes^3^. From clinical risk factors to ‘omics’ technology, there is still currently no single good predictor of preeclampsia^10–15^.

Clinical factors remain an inexpensive and rapid way to predict the occurrence of preeclampsia. This current study intends to evaluate the incidence of preeclampsia and its sub-phenotypes (early-onset and late-onset), socio-demographic and clinical risk factors for preeclampsia, as well as assess the ability to predict this disorder in a cohort of healthy nulliparous Brazilian pregnant women.

## Results

**Figure 1:**
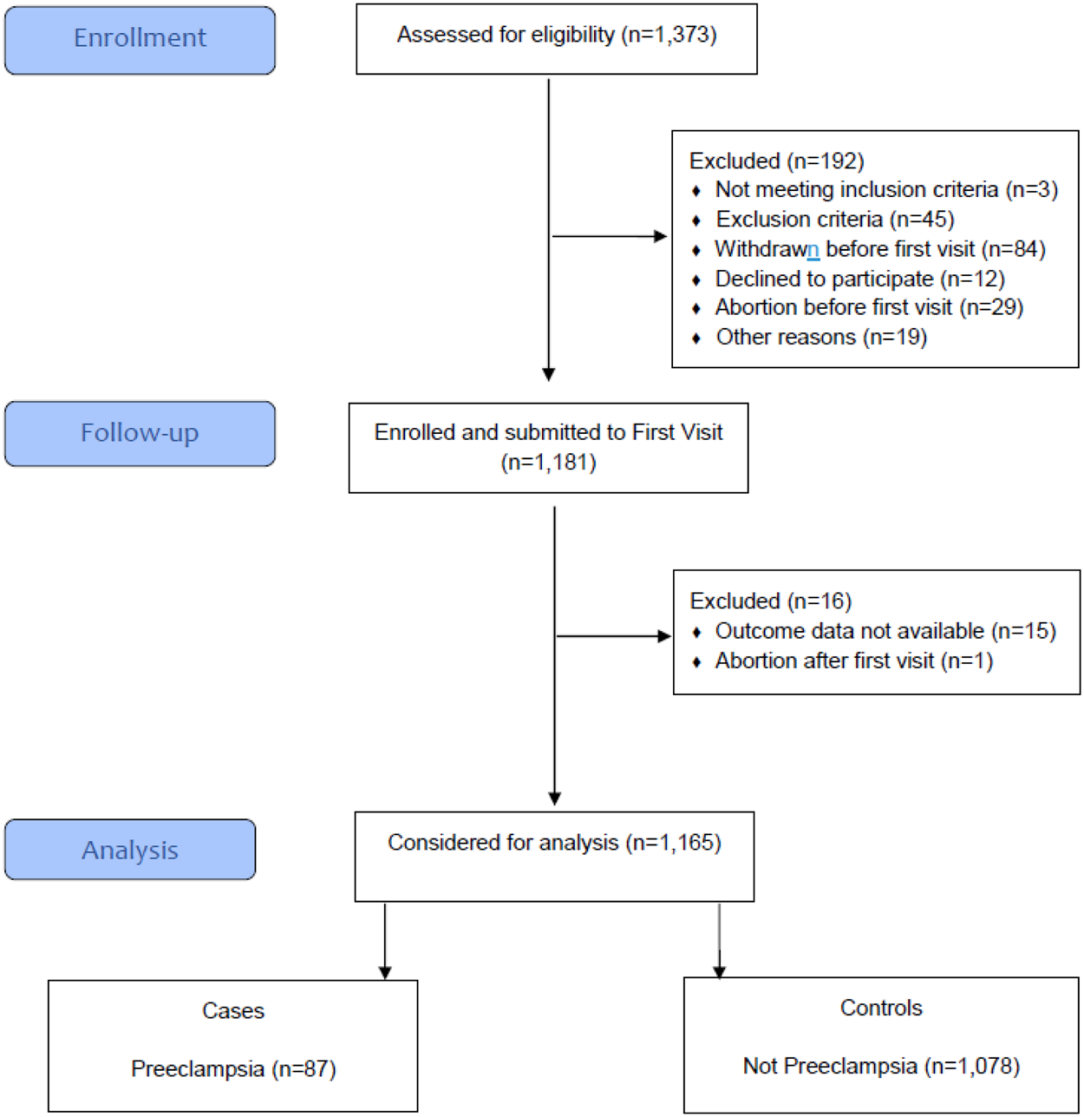
Flowchart of women participating in the study.

Among 1,373 participants screened for eligibility in the Preterm SAMBA study, complete pregnancy outcome data were available for 1,165 women (Fig. 1). Preeclampsia developed in 87 (7.5%) participants of whom 14 (16.1%) had early-onset preeclampsia while the remaining 73 were late-onset. The socio-demographic characteristics of women who developed preeclampsia and controls are shown in Table 1. Among patient characteristics, the rate of weight gain per week equal to or more than 0.75 kg, obesity (BMI > 30.9 Kg/m^2^) and diastolic blood pressure equal to or higher than 75 mmHg at 20 weeks of gestation were associated with more than twice the risk of preeclampsia (Table 1).

**Table 1:** Estimated risk of selected socio-demographic and some medical history and personal characteristics in preeclampsia.

Characteristics	Preeclampsia	Controls	RR (95% CI)
**Maternal age (years)**	**n** (%)	**n** (%)	
<20	20 (23)	271 (25.1)	0.91 [0.48–1.74]
20–34	60 (69)	736 (68.2)	Ref.
>34	7 (8)	71 (6.7)	1.19 [0.50–2.85]
**Ethnicity**			
White	27 (31)	435 (40.3)	Ref.
Others	60 (69)	643 (59.7)	1.46 [0.84–2.55]
**Marital status** ^a^			
With partner	63 (72.4)	777 (72.0)	Ref.
Without partner	24 (27.6)	296 (27.4)	1.00 [0.47–2.12]
**Schooling (years)**			
<12	58 (66.6)	733 (68.0)	Ref.
≥12	29 (33.4)	345 (32.0)	1.06 [0.45–2.51]
**Annual Family Income (US$)**			
Up to 3000	24 (27.6)	280 (25.9)	1.18 [0.63–2.23]
3000 to 6000	31 (35.6)	350 (32.4)	1.22 [0.64–2.34]
Above 6000	32 (36.8)	448 (41.7)	Ref.
**Source of prenatal care**			
Entirely public	81 (93.1)	927 (85.9)	2.10 [0.50–8.93]
Private/insurance/mixed	6 (6.9)	151 (14.1)	Ref.
**Family history of hypertensive disease**			
Any hypertensive disorder^b^	16 (18.4)	141 (13.0)	1.47 [0.83–2.60]
Pregnancy of participant’s mother	4 (4.6)	43 (3.9)	1.15 [0.12–10.62]
**Smoking**			
No smoking	81 (93.1)	997 (92.4)	Ref.
Stopped during pregnancy/current smoker	6 (6.9)	81 (7.5)	0.92 [0.18–4.58]
**Use of illicit drugs** ^**c**^			
Non-user	68 (78.2)	873 (80.9)	Ref.
Ceased during pregnancy/current user	2 (2.3)	52 (4.8)	0.51 [0.22–1.22]
**Weight gain rate per week (kg)** ^**d**^			
<0.25	14 (16.1)	127 (11.7)	1.67 [0.42–6.59]
0.25–0.49	23 (26.4)	364 (33.7)	Ref.
0.50–0.74	26 (29.9)	367 (34.0)	1.11 [0.70–1.77]
≥0.75	15 (17.2)	109 (10.1)	**2.04 [1.12–3.69]**
**Body Mass Index at enrolment** ^**e,&**^			
Underweight (<21.5 kg/m^2^)	9 (10.3)	190 (17.6)	0.74 [0.38–1.45]
Normal weight (21.5–26.2)	28 (32.2)	433 (40.1)	Ref.
Overweight (26.3–30.9)	22 (25.3)	280 (25.9)	1.20 [0.75–1.92]
Obesity (>30.9)	28 (32.2)	174 (16.1)	**2.28 [1.39–3.74]**
**Any previous maternal conditions** (anaemia, thyroid, asthma, previous hypertensive disorder*, depression, POS)			
No	53 (60.9)	730 (67.7)	Ref.
Yes	34 (39.1)	348 (32.3)	1.31 [0.66–2.60]
**Diastolic pressure at 20 weeks’ gestation** ^**e**^			
<75 mmHg	64 (73.6)	937 (86.9)	Ref.
≥75 mmHg	23 (26.4)	140 (12.9)	**2.21 [1.30–3.74]**
**Total**	**87**	**1078**	

Missing information for ^a^5; ^b^100; ^c^170; ^d^120; ^e^1case; ^#^RR and 95% CI not presented due to small numbers; values in bold mean they are significant.

*Without using medication.

& ref.^41^.

Maternal and neonatal outcomes in preeclampsia were worse for both the mothers and their neonates (Table 2). Women with preeclampsia presented a relative risk of 3.58 for caesarean section, while hospital admission for 5 days or more was almost 6-fold higher. Women with preeclampsia had more preterm births at less than 34 weeks of gestation (3.97 fold) than controls. Neonates of women with preeclampsia had a significantly lower birthweight (a mean of 379 g lower), and there was a twofold to threefold higher occurrence of small for gestational age (SGA) babies, 5-minute Apgar scores less than 7, NICU admission and Neonatal Near Miss events. There was only one case of fetal death, occurring in a 26-year old woman, at 26 weeks of gestation. She was admitted to hospital, complaining of a headache. Arterial blood pressure was 170 × 110 mmHg, protein in dipstick urinalysis was +3 and no fetal heart beat was identified. The induction of labour lasted 24 hours, resulting in vaginal delivery of a baby weighing 620 g.

**Table 2:** Maternal and neonatal outcomes associated with preeclampsia.

Characteristics	Preeclampsia n (%)	Controls n (%)	RR (95% CI)
**Mode of delivery**			
Vaginal	21 (24.1)	599 (55.5)	Ref.
C-section	66 (75.9)	479 (44.5)	**3.58 [1.57–8.12]**
**Onset of labour**			
Spontaneous	18 (20.7)	664 (61.5)	Ref.
Induced	36 (41.4)	211 (19.5)	**5.52 [2.21–13.83]**
Elective C-section	33 (37.9)	203 (19.0)	**5.30 [1.25–22.38]**
**Gestational age at birth (weeks)**			
<34	11 (12.6)	32 (3.0)	**3.97 [1.55–10.20]**
34–36	9 (10.3)	73 (6.7)	1.70 [0.57–5.06]
≥37	67 (77.1)	973 (90.3)	Ref.
**Length of postpartum hospitalization**			
1–4 days	67 (77.0)	1041 (96.5)	Ref.
≥5 days	20 (23.0)	37 (3.5)	**5.80 [2.12–15.91]**
**Thromboembolic event before or after delivery**			
No	87 (100.0)	1072 (99.4)	Ref.
Yes	0	4 (0.4)	#
**Mean (SD) birthweight (g)**	2779.4 (±843.1)	3158.8 (±558.9)	*WMD = **−379.4 (−644.5 to −114.4)**
**Adequacy of birthweight to GA** ^**a**^			
SGA (p < 10)	22 (25.3)	124 (11.5)	**2.45 [1.52–3.95]**
AGA (10 < p < 90)	54 (62.0)	824 (76.4)	Ref.
LGA (p > 90)	10 (11.5)	118 (10.9)	1.27 [0.76–2.13]
**Fetal death**	1 (1.1)	6 (0.5)	1.92 [0.09–39.42]
**Apgar score – at 5 minutes** <**7**^**b**^	3 (3.4)	16 (1.5)	**2.11 [1.03–4.29]**
**Intubation required after birth**	7 (8.0)	19 (1.7)	3.89 [0.41–36.95]
**NICU admission**	32 (36.7)	141 (13.1)	**3.34 [1.61–6.90]**
**Neonatal Near Miss (Apgar 5 < 7 OR Birthweight <1750 g OR GA <33)** ^**c**^	13 (14.9)	39 (3.6)	**3.65 [1.78–7.49]**
**Total**	**87**	**1078**	

Missing information for ^a^13 cases; ^b^65 cases; ^c^62 cases. Values in bold mean they are significant.

^#^RR and 95% CI not presented due to small numbers.

*WMD: weighted mean difference.

On multivariate analysis, diastolic blood pressure at 20 weeks of gestation and BMI at enrolment were independently associated with the occurrence of preeclampsia, with an adjusted risk ratio of 1.04 (Table 3).

**Table 3:** Factors independently associated with preeclampsia on multivariate analysis [n = 1164].

Characteristics	RR_adj_ (95% CI)
Diastolic blood pressure at 20 weeks’ gestation (mmHg)	1.04 [<1.01–1.06]
Body Mass Index at enrolment (kg/m^2^)	1.04 [1.01–1.09]

Variables included in the model (14): Maternal age (years); Ethnicity (White: 0/other: 1); Marital status (with partner: 0/without partner: 1); Schooling (<12 years: 0/≥12 years: 1); Annual Family Income (Up to US$6000: 1/>US$6000: 0); Source of prenatal care (entirely public: 1/other: 0); Family history of hypertensive disease: Any hypertensive disorder (yes: 1/no: 0); Pregnancy of participant´s mother (yes: 1/no: 0); Smoking (yes: 1/no smoking: 0); Use of illicit drugs (yes: 1/non-user:0); Weight gain rate per week (kg); Body Mass Index at enrolment (kg/m^2^); Any previous maternal conditions (yes: 1/no: 0); Diastolic blood pressure at 20 weeks’ gestation (mmHg).

## Discussion

Our study revealed that the incidence of preeclampsia was 7.5% in a nulliparous group of healthy pregnant women from three different Brazilian regions, which is higher than values obtained from other cohorts of nulliparous pregnant women^16–18^. Current analysis was able to identify only three factors significantly associated with the development of preeclampsia: weight gain rate per week, obesity and value of diastolic blood pressure measured at 20 weeks of gestation equal to or higher than 75 mmHg. The low number of preeclampsia cases in this sample probably prevented us from identifying additional factors, limiting the capacity to predict preeclampsia by using a composition of factors. Not surprisingly, our findings on maternal and perinatal outcomes added support to other studies, showing an increased frequency of Caesarean sections, preterm births, neonatal near misses, 5-minute Apgar scores less than 7 and low birth weight in pregnancies complicated by preeclampsia^19,20^. This higher proportion of adverse perinatal outcomes, including the lower birthweight, are also related to the increased occurrence of preterm births among preeclamptic women.

To the best of our knowledge, this was the first time that a Brazilian cohort of healthy nulliparous pregnant women received follow-up with data acquisition on the incidence of preeclampsia. The manner of calculating the rate of weight gain was a limitation of our analysis. Patients were recruited from 19 to 21 weeks of gestation to the last measurement at the end of prenatal care. Owing that the last time measure was done between 37 and 39 weeks of gestation, a quarter of preeclampsia cases (22.9%) were not included. Potential bias – reverse causality – may occur, since after preeclampsia is diagnosed, weight is influenced by oedema, a potential feature of this disease. Another limiting characteristic is that the database does not have information on the precise time when antihypertensive drugs (if used) were initiated.

In our cohort, data on the actual incidence of preeclampsia was totally distinct from findings of a systematic review published in 2008 showing a prevalence of 1.5% for preeclampsia and 0.6% for eclampsia. According to those authors, their numbers were underestimated in some regions due to lack of information^8^. Almost 10 years later, a study implemented in Brazil showed that the prevalence of preeclampsia was 8.1% in specific regions^21^. Our study currently revealed that the incidence of preeclampsia is 7.5% in a nulliparous group of healthy pregnant women, which is higher than values obtained from other cohorts^16–18^. The high prevalence of obesity in our population may explain the incidence of preeclampsia. Despite the lack of data available on this topic in our country, a recent cross-sectional study involving 1,279 pregnant women showed that the prevalence of overweight or obesity during the first prenatal visit was almost 40%^22^.

Owing to the high incidence and the potential impact of preeclampsia^2,23,24^, it is essential to find an effective tool that provides early identification of pregnant women at high risk for this disease, in order to implement prophylactic measures and avoid harmful consequences. A search for a predictive model with widespread global applicability has begun, encouraged by the results achieved by studies using low-dose aspirin as a prophylactic measure^25,26^. However, the prediction of preeclampsia is challenging, in view of the complexity of its aetiology^27^. It is unlikely that a single risk factor will be able to predict the occurrence of this condition. In addition, our results come from women that were initially screened at around 20 weeks, after the period when some known prophylactic measures are recommended to be started.

Maternal clinical factors have emerged as an interesting screening alternative. In 2010, a guideline of the National Collaborating Centre for Women’s and Children’s Health recommended the use of maternal clinical factors as screening tests. According to the guideline, a previous history of gestational hypertensive disorder, autoimmune disease (systemic erythematous lupus or antiphospholipid syndrome), chronic renal disease, diabetes and chronic hypertension are considered high-risk factors. Once any of these factors are present, prophylactic measures must be initiated^28^. The NICE screening proposal was assessed in a prospective study involving a heterogeneous population composed of nulliparous and multiparous pregnant women. Detection rates of 37% and 28.9% were obtained for early-onset (before 34 weeks of gestation) and late-onset (at or after 34 weeks of gestation) preeclampsia cases, respectively^29^. These numbers were confirmed in another study applying the NICE criteria with a third of preeclampsia cases identified^30^.

Our cohort identified only three factors related to increased risk of preeclampsia: weight gain rate per week, obesity and value of diastolic blood pressure measured at 20 weeks of gestation equal to or higher than 75 mmHg. A cohort of more than 62,000 nulliparous pregnant women generated the same finding concerning the influence of weight gain per week on preeclampsia risk^18^. We also reinforced previous evidence that obesity predisposes to the occurrence of preeclampsia, especially in late-onset cases. This is probably associated with the inflammatory property of adipose tissue and its effects on endothelial function^31^. Considering that both BMI and weight gain rate are modifiable risk factors, consolidated knowledge of their predictive function emphasises the importance of antenatal counselling and prenatal care follow-up^32,33^.

Our cohort of nulliparous healthy pregnant women also indicated that a diastolic blood pressure higher than 75 mmHg was correlated with the occurrence of preeclampsia. This finding conflicts with another study^34^, which showed that mean arterial blood pressure was a better predictor of preeclampsia in a healthy group of pregnant women.

The modest predictive power achieved through models with only maternal clinical factors have prompted prospective studies among heterogeneous populations. These studies used multivariate analysis combining maternal clinical factors to other elements such as uterine artery Doppler and biomarkers, in order to develop algorithms of preeclampsia prediction. Although the detection rates of the resulting algorithms were high, these studies were implemented among a heterogeneous population of pregnant women at high risk of developing preeclampsia^29,30^. Furthermore, these studies did not segregate nulliparous women, which is a limitation. It is well-known that the most consistent predictive clinical factor for preeclampsia, which is a previous history of preeclampsia, cannot be applied to first-time mothers^3^.

Biochemical factors have been studied, with modest results in terms of prediction potential^35,36^. Furthermore, the potential costs incurred and technologies available for biomarker processing represent a limiting factor for use on a large scale, especially in low and middle-income countries. Thus, clinical risk factors continue to play a crucial role as a cheap and tangible screening instrument for preeclampsia.

To date, no single screening test has shown sufficiently accurate specificity and sensitivity to predict preeclampsia cases^37^. The value of clinical risk factors, biochemical markers, uterine Doppler as predictive markers addressed separately remains modest at best for all women destined to develop preeclampsia^38^. This is probably due to the multifactorial aetiology of the condition. Genetic, immunological, environmental and maternal factors have all made their contributions and remain to be fully elucidated. Therefore, preeclampsia is a heterogeneous disease concerning clinical presentation, pathology and outcomes. Notwithstanding decades of research, an enigma still persists surrounding a useful and accurate screening test model to identify early on pregnant women, primarily in the nulliparous group, at high risk for preeclampsia. Research into this field is of considerable importance.

## Methods

This is a nested case-control study derived from a secondary analysis of the Preterm-SAMBA study (Preterm Screening and Metabolomics in Brazil and Auckland), a prospective multicentre cohort study conducted in five Brazilian centres between July 2015 and March 2018. The research protocol was previously published elsewhere^39^. Briefly, the original study design was based on the primary goal of developing a predictive model for preterm birth. The study was developed in two phases: a discovery phase and a validation phase. The first phase was a case-control study, involving participants from the previously described SCOPE study^3^. In the validation phase, a prediction model was validated in the Preterm SAMBA Brazilian cohort. Other maternal and perinatal outcomes of interest were considered as secondary objectives included preeclampsia (currently addressed), gestational diabetes mellitus and fetal growth restriction. For this nested case-control approach, cases were women who developed preeclampsia and controls were all the remaining women free from the disorder. Preterm-SAMBA study was conducted according to Declaration of Helsinki guidelines. Appropriate approval was obtained from the five centres involved in the study. All recruited participants gave their written informed consent.

### Participants

The study enrolled healthy nulliparous pregnant women between 19 and 20 + 6 weeks of gestation, with a singleton pregnancy, from five different centres in Brazil (from Campinas, Botucatu, Recife, Fortaleza and Porto Alegre). Exclusion criteria were: 3 or more previous abortions; cervical suture; fetal malformation; chronic hypertension requiring antihypertensive drugs and/or diabetes and/or renal disease; arterial blood pressure higher than 160 × 100 mmHg at the time of enrolment; Systemic Lupus Erythematosus and/or antiphospholipid syndrome; sickle cell disease; HIV infection; congenital uterine anomalies (bicornuate uterus, septate uterus); previous cervical knife cone biopsy; chronic exposure to corticosteroids or calcium at a dosage above 1 g or fish oil at a dosage above 2.7 g per day or vitamin C above 1000 mg per day or vitamin E above 400 UI per day; heparin or aspirin use (any dosage or presentation form). Our inclusion and exclusion criteria were decided to be aligned with another study, previously published called SCOPE^3^ that used exactly the same criteria. This is the reason why obesity was not considered exclusion criteria, although for some authors it is considered a major risk factor for preeclampsia^27^.

### Sample size estimation

Sample size was calculated according to the primary outcome - preterm birth. Assuming a type I error of 5% and accuracy of the test of at least 0.68 according to the area under the ROC curve, and to test the hypotheses with adequate power (80% of power, β = 0.2), the sample size would need to approach 80 cases of preterm delivery. The minimum expected prevalence of this outcome was presumed to be 7% in Brazil, therefore the sample size was calculated at 1150 women. In addition, considering that the mean prevalence of preeclampsia observed in larger studies of around 5–6%^8,17^ among nulliparous women, it was anticipate that this cohort would incorporate around 58 to 69 cases of preeclampsia.

### Procedures

All steps of the main study have been previously described^39^. Data were collected at three different set points (visits) during follow-up. On the first visit, between 19 and 21 weeks of gestation, a full assessment was performed to gather information on sociodemographic characteristics, reproductive family history, current or previous diseases, personal habits, with a complete follow-up until delivery and immediate postpartum period. During the interview, data were entered into a central database with internet access and complete audit trail (MedSciNet). Anthropometric measurements plus nutritional assessment were also performed. The same evaluation was conducted on both subsequent visits, at 27–29 weeks of gestation and at 37–39 weeks of gestation.

### Outcome

The outcome of interest for the current analyses is preeclampsia. In this study, preeclampsia was defined as the occurrence of hypertension (SBP ≥ 140 and/or DBP > 90 mmHg) in at least two different time periods, combined with proteinuria (300 mg/24 hour or at least 1 g/L [2+] on dipstick testing or spot urine protein/creatinine >30 mg/mmol [0.3 mg/mg]). Preeclampsia was also classified as early-onset when diagnosed before 34 weeks of gestation and as late-onset otherwise. In the absence of proteinuria, the disorder was also defined as the occurrence of any systemic complications/organ dysfunction^40^ including:

- Haematological complications (thrombocytopenia - platelet count below 100,000/dL, DIC, haemolysis);
- Hepatic dysfunction (elevated liver enzymes – at least twice the upper limit of normal + right upper quadrant or epigastric abdominal pain);
- Neurological dysfunction (eclampsia, altered mental status, blindness, stroke, hyperreflexia with clonus, severe headaches, visual scotomata when persistent);
- Renal dysfunction (creatinine ≥1.2 mg/dL).

### Statistical analysis

We determined the general incidence of preeclampsia and early-onset and late-onset preeclampsia. Several socio-demographic, clinical factors and lifestyle habits were regarded as potential risk factors. Furthermore, maternal and neonatal outcomes associated with preeclampsia were addressed. Bivariate analysis was performed, estimating the Risk Ratios (RR) and their respective 95% Confidence Intervals, using *Student’s t*, chi-square or Fisher’s exact tests accordingly. Finally, a multivariate analysis with a Poisson regression model was performed to identify which factors were independently associated with preeclampsia in this sample, estimating the adjusted RR for those identified. Each centre/hospital was considered as a Primary Sampling Unit (PSU) in every analysis. SPSS software version 20.0 and Stata software version 7.0 were used for analysis.

### Ethical considerations

The current study is an ancillary analysis (preeclampsia) of the outcome from a Brazilian cohort of low-risk nulliparous women entitled “Preterm SAMBA” which was financially supported by the Bill and Melinda Gates Foundation and the Brazilian CNPq. The Preterm SAMBA study has been reviewed and approved by the Brazilian National Committee for Ethics in Research (CONEP) and by the Institutional Review Board (IRB) of the coordinating centre (Letter of approval 1.048.565 issued on 28th April 2015) and of all other Brazilian participating centres. Before enrolment, each woman was full explained about the study and signed an informed consent form.

## Data Availability

The datasets generated and analysed during the current study are available from the corresponding author on reasonable request. The participating women did not give their consent to make their own data publicly available.
